# Genome Sequence of the Oleaginous Yeast *Rhodotorula glutinis* ATCC 204091

**DOI:** 10.1128/genomeA.00046-14

**Published:** 2014-02-13

**Authors:** Debarati Paul, Zenaida Magbanua, Mark Arick, Todd French, Susan M. Bridges, Shane C. Burgess, Mark L. Lawrence

**Affiliations:** a Amity Institute of Biotechnology, Amity University, AUUP, Noida, India; b Institute for Genomics, Biocomputing, and Biotechnology, Mississippi State University, Mississippi State, Mississippi, USA; c Dave C. Swalm School of Chemical Engineering, Mississippi State University, Mississippi State, Mississippi, USA; d Department of Computer Science and Engineering, Mississippi State University, Mississippi State, Mississippi, USA; e College of Agriculture and Life Sciences, University of Arizona, Tucson, Arizona, USA; f College of Veterinary Medicine, Mississippi State University, Mississippi State, Mississippi, USA

## Abstract

*Rhodotorula glutinis* ATCC 204091 is an oleaginous oxidative red yeast that can accumulate lipids to >50% of its biomass when grown with appropriate carbon and nitrogen ratios. It produces a red pigment consisting of useful antioxidants, such as carotenoids, torulene, and torularhodin, when cultivated under carbon-deficient conditions.

## GENOME ANNOUNCEMENT

*Rhodotorula glutinis* is an oleaginous oxidative red yeast that potentially can be used for biodiesel production as well as other important products, such as carotenoids (torulene, torularhodin, γ-carotene, and β-carotene) when grown on suitable culture medium containing appropriate carbon and nitrogen ratios (1–3). Historically, *R. glutinis* has been used to identify a yeast pathway for l-lysine biosynthesis via l-pipecolic acid (4). *Rhodotorula* secretes α-l-arabinofuranosidase, which is important to the wine industry for aroma of the wine. *R. glutinis* is nonfastidious, making it attractive for the production of phenylalanine ammonia lyase (PAL) (EC 4.3.1.5), which is a nonhydrolytic enzyme produced in high quantities by *R. glutinis* (5). Here, we report the *R. glutinis* genome sequence, which can be used to study the regulation of these important pathways and to explore genes encoding other useful products.

Genomic DNA was isolated using the Wizard genomic DNA purification kit (Promega) with overnight lyticase treatment (20 mg/ml lyticase [catalog no. L2524; Sigma]) to lyse the cells. The purity and the concentration of DNA were measured by NanoDrop (Thermo Scientific). Genome sequencing was conducted using a 454 GS-FLX Titanium platform. One standard run and one paired-end run were completed, resulting in a genome size of 20,476,699 bp that was organized in 384 large contigs (≥500 bp) and 29 scaffolds. Approximately 200 scaffolded gaps were amplified by PCR, and subsequent Sanger sequencing closed 176 gaps, resulting in an assembly with 208 contigs. An Illumina sequencing run was also conducted on genomic DNA from *R. glutinis* and assembled with our previous 454 and Sanger data using CAP3 and SSAKE, resulting in an assembly containing 186 contigs in 29 scaffolds.

Expressed sequence tags (ESTs) are not available to assist in annotation. Therefore, GeneMarkS (6), a self-training *ab initio* gene finder, was used for the initial gene prediction. This set of predicted genes was used to train Augustus (7) and Glimmer (8). A comprehensive gene prediction run was performed on the entire genome using GeneMark, Augustus, and Glimmer. RepeatMasker was used to screen for repeats and low-complexity sequences. BLAST searches were conducted on predicted genes against the nonredundant NCBI database. Based on these results, annotations were manually curated and evaluated using InterPro (9). To aid in the manual annotation, Apollo was used to view the predicted gene models and repetitive elements.

The *R. glutinis* genome is 20,478,880 bp, with a G+C content of 61.9%. The length of putative genic regions is 17,229,291 bp, and there are 3,359 putative genes coding for 2,817 proteins. By comparison, the genome of the systematically similar yeast *Ustilago maydis* 521 is 19,742,445 nucleotides (nt) in length and has a 54% G+C content with 6,631 genes. The *R. glutinis* genome sequence will enable pathway analysis and functional genomics investigations, which will allow for the identification of regulatory mechanisms controlling lipid accumulation. By improving the efficiency of carotenoid/lipid accumulation in *R. glutinis*, this yeast has the potential to produce useful antioxidants and biodiesel.

### Nucleotide sequence accession numbers.

The *R. glutinis* whole-genome shotgun (WGS) project (PID-59971) has been deposited at DBJ/EMBL/GenBank under the accession no. AEVR00000000 and consists of (WGS) sequences AEVR02000001 to AEVR02000029. The version described in this paper is AEVR02000000.
